# Analysis of Aggregate Morphological Characteristics for Viscoelastic Properties of Asphalt Mixes Using Simplex Lattice Design

**DOI:** 10.3390/ma11101908

**Published:** 2018-10-08

**Authors:** Wensheng Wang, Yongchun Cheng, Guojin Tan, Jinglin Tao

**Affiliations:** 1 College of Transportation, Jilin University, Changchun 130025, China; wangws17@mails.jlu.edu.cn (W.W.); chengyc@jlu.edu.cn (Y.C.); 2 Jiangxi Transportation Institute, Nanchang 330200, China

**Keywords:** asphalt mixes, aggregate characteristics, simplex lattice design, viscoelastic properties

## Abstract

Morphological characteristics of aggregates have direct impacts on performances of asphalt mixes. This paper aims to investigate the effects of the morphological characteristics of fine and coarse aggregates on the high-temperature viscoelastic properties of asphalt mortars and mixtures. For this purpose, an experimental proportion scheme was designed for asphalt mixes prepared with three different types of aggregates (basalt, andesite and pebble/river sand) based on the simplex lattice design (SLD) method. Three morphological parameters were chosen to characterize shape, angularity and texture of aggregates. Afterwards, the uniaxial compression creep test was conducted for asphalt mixes and the high-temperature viscoelastic properties were obtained based on Burgers model. The effects of fine and coarse aggregates on the viscoelastic properties are analyzed through asphalt mortars and mixtures, respectively. The results showed that aggregate morphological characteristics correlate with the high-temperature viscoelastic properties of asphalt mixes, especially for fine aggregates. Aggregates with complex morphological characteristics are conducive to improving the deformation recovery and anti-deformation of asphalt mixes. Furthermore, coarse aggregates can enhance the anti-deformation of asphalt mixture effectively due to its skeleton effect.

## 1. Introduction

Asphalt as a kind of composite material has been widely used in pavement and building constructions. In general, aggregates in asphalt mixtures account for approximately over 90% of mixtures by weight, which play a major role in its stability, durability and mechanical properties. Researchers in many countries have been trying to focus on the influence of aggregate morphological characteristics on performances of asphalt mixture [1,2,3].

A great number of laboratory tests have been developed in previous research to evaluate the morphological characteristics of aggregates. Wilson and Klotz [4] presented a method of measuring angularity using Hough transform for quantitative analysis of aggregate angularity. Kuo et al. [5] used a digital image-analysis method to investigate the morphologies of coarse aggregates and effectively quantified the morphological characteristics. The morphological characteristics were found to be correlated well with indirect characterization test results by regression analysis. Wang et al. [6] developed a unified Fourier morphological analysis method to quantify aggregate morphological characteristics, which include shape, angularity and surface texture. The above analysis has been conducted on coarse aggregates; however, investigations on fine aggregate morphology have attracted more researchers. Masad and Button [7] investigated the angularity and texture of fine aggregates by using the erosion-dilation method and form factor, in which angularity is analyzed by high-resolution images and texture is described by low-resolution images. Kuo and Freeman [8] defined three image indices—aspect ratio, angularity and roughness—to characterize overall shape, roundness of corners, and particle surface texture of fine aggregates, which could be calculated by shortest and longest dimensions, perimeters, convex perimeters and perimeters of ellipses. Xie et al. [9] evaluated the morphological characteristics of fine aggregates by using three methods and they found that different methods have respective applicability and precision, in which digital image processing technique is useful for designing bituminous materials. Xiao et al. [10] studied fine aggregate morphology by using the aggregate image measurement system and discussed the influences of aggregate morphological characteristics on skid-resistance of single-grade micro-surfacing.

Based on previous research on aggregates’ morphological characteristics, the relationships between aggregate morphological characteristics and performances of asphalt mixtures were also investigated. Petersen et al. [11] aimed at evaluating the rutting resistance of asphalt mixture and found that the morphological characteristics of coarse aggregate correlated well with it. Arasan et al. [12] used the digital image processing method to analyze the shape indices of coarse aggregate and showed a good correlation between some shape indices of aggregate and volumetric performances of asphalt mixtures. Singh et al. [13] utilized aggregate shape parameters to estimate dynamic modulus of asphalt mixes by establishing a model. Pan et al. [14] investigated the effects of coarse aggregate morphology on the permanent deformation of asphalt mixtures. The permanent deformation showed a strong correlation with surface texture and angularity; the former has significant influences on the permanent deformation. Aragao et al. [15] evaluated the influence of morphological properties of aggregates on the mechanical behavior of bituminous mixtures. The results indicated that the morphological characteristics of coarse aggregate are strongly correlated with the resistance to rutting of asphalt mixtures. Besides, the aggregate surface texture was proven to be highly correlated to the performance of mixtures and should be carefully considered in aggregate and asphalt mixture specifications. Valdes-Vidal et al. [16] investigated the influence of aggregate physical properties on the mechanical properties of asphalt concretes. Morphological characteristics as well as surface texture have been conducted for three aggregates. The results demonstrated that the morphological characteristics of coarse and fine aggregates influence strength and anti-cracking properties of asphalt concretes, which depend on the shredding process and the origin of aggregates. Masad et al. [17] addressed the relationship between the morphological characteristics of fine aggregate and performances of asphalt mixtures and they found that texture characteristics had the strongest correlation with rutting resistance of asphalt mixtures. Therefore, the morphological characteristics of coarse and fine aggregates have significantly different influences on the performances of asphalt mixture.

Morphological characteristics of aggregates have direct impacts on performances of asphalt mixtures. Previous research has investigated the influences of aggregate morphological characteristics on the adhesion between the aggregate and asphalt, high- and low-temperature stability performances, and fatigue property. However, not much study has been conducted on the relationship between an aggregate’s morphological characteristics and viscoelastic properties of the asphalt mixture. Therefore, in order to investigate the influence of an aggregate’s morphological characteristics on asphalt mixture, this paper studied the effects of the morphological characteristics of fine and coarse aggregates on the high-temperature viscoelastic properties of asphalt mortars and mixtures.

In this paper, an experimental proportion design was created for asphalt mortars and mixtures prepared with three different types of aggregates, based on the simplex lattice design (SLD) method. Three indices are chosen to characterize aggregates—shape, angularity and texture. The objective is to relate the aggregate’s morphological characteristics to the viscoelastic properties of asphalt mixture. Based on Burgers viscoelastic model, the uniaxial compression static creep test was carried out to analyze the high-temperature viscoelastic properties of asphalt mortar and mixture.

## 2. Materials and Methods

### 2.1. Raw Materials

In this study, asphalt AH-90 from Panjin Petrochemical Industry of Liaoning Province, China, was chosen for the asphalt mixes. Table 1 shows the physical properties of AH-90. The mineral fillers and aggregates were obtained from a local stone factory in Jilin Province, China. The selected filler is ordinary limestone powder, and three types of aggregates with various morphological characteristics were chosen for coarse and fine aggregates, respectively. The three types of coarse aggregates were basalt stone, andesite stone and pebble; fine aggregates included basalt manufactured sand, andesite manufactured sand and river sand. Their physical properties are summarized in Table 2.

### 2.2. Sample Preparation

**Figure 1:**
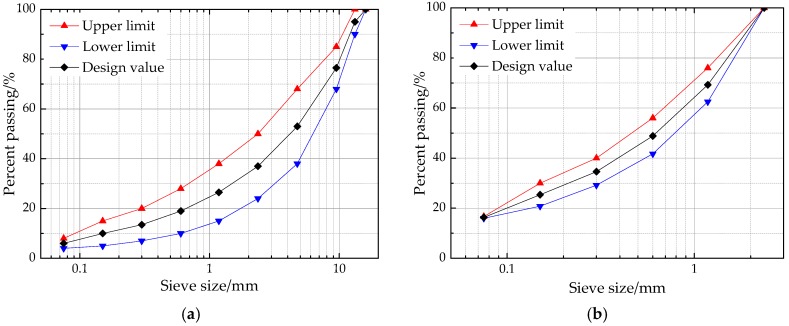
Gradations of asphalt mixes used in this study: (**a**) asphalt mixture; (**b**) asphalt mortar.

Asphalt mixes (asphalt mortar and asphalt mixture) were prepared to analyze and demonstrate the influence of coarse and fine aggregates characteristics on high-temperature viscoelastic properties. Figure 1 presents the used gradations of asphalt mixes with a nominal maximum size of 13.2 mm, i.e., AC-13, in which asphalt mortar consists of asphalt, filler and fine aggregate passing through 2.36 mm [18]. Besides, asphalt content is generally regarded as one of the main influencing factors for asphalt mixes. Its consistency is of great importance. According to previous literature [18], asphalt film thickness was kept as a constant of 8 μm and the specific surface area of asphalt mixes was calculated as 14.34 m^2^/kg for asphalt mortar and 5.935 m^2^/kg for asphalt mixture. Thus, the optimal asphalt content can be also obtained as 11.6% for asphalt mortar and 4.8% for asphalt mixture. The target air void of asphalt mixes was set as 4% and then the mass and volume proportion of asphalt mixes can be determined through the densities of asphalt and aggregates. The cylindrical specimens of asphalt mortar with height of 50 mm and diameter of 50 mm were prepared through the static pressure method [19]. The Marshall specimens of asphalt mixture with height of 63.5 mm and diameter of 101 mm were prepared through Marshall procedures, according to Chinese specification JTG E20-2011 [20].

### 2.3. Experimental Methods

#### 2.3.1. Morphological Characteristics of Aggregates

A scanner (Opt Vision Technology Co., Ltd., Dongguan, China) is selected to obtain the image information of coarse aggregates, and a stereo microscope (Opt Vision Technology Co., Ltd.) is used to collect the image information of fine aggregate. Before image collection, fine and coarse aggregates need to be cleaned to remove impurities (such as surface dust) and then dried in an oven. The image processing technique is then employed to process (image denoising and enhancement) the color images. Subsequently, the morphological characteristics of aggregates are obtained through the following equations.

(1) Shape

Shape characteristics of aggregates reflect flat and elongated particles, which are undesirable in the preparation of asphalt mixture because they break easily under loading effects. Roundness is used to represent the shape of aggregates, which could be calculated as follows [17]:

*R* = *L*^2^/4π*S* (1)

where *R* is shape index, *L* is the projected perimeter of the particle, *S* is the corresponding projected area. In general, *R* ≥ 1, a larger *R* value means a slender shape and the aggregate is more spherical if the *R* value is closer to one.

(2) Angularity

Angularity of the aggregates illustrates the angle change on particle outline, and perimeter index expressed in Equation (2) is used to characterize the angularity characteristics of aggregates [21].

*PI* = *P*/*P_E_* (2)

where *PI* is perimeter index, *P* is the perimeter of particle outline, *P_E_* is the perimeter of equivalent ellipse. The larger *PI* means more angular boundaries or sharp angles of the aggregate.

(3) Texture

Texture is usually considered as the tiny bumps on the particle outline [21]. In this study, the erosion-dilation area ratio is regarded as an evaluation index for texture characteristics of aggregates, which could be expressed as [7]:

*EDR* = (*A*_1_ − *A*_2_)/*A*_1_ × 100 (3)

where *EDR* is texture index, *A*_1_ is area of the original particle, *A*_2_ is area of the particle after successive erosion and dilation operations.

#### 2.3.2. Burgers Viscoelastic Model

**Figure 2:**
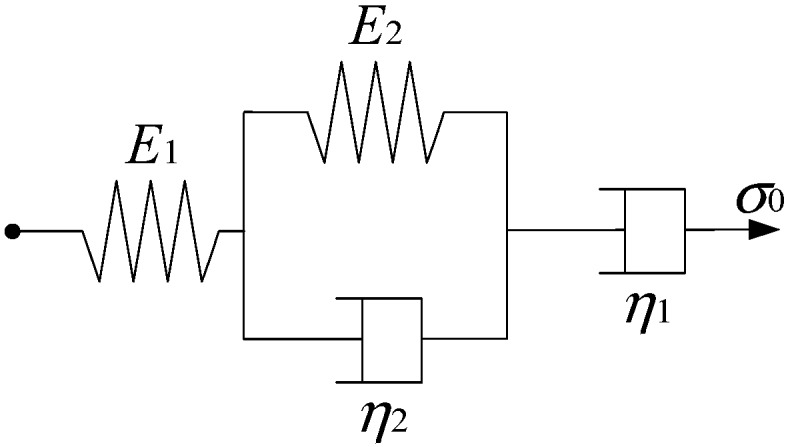
Schematic representation of Burgers model.

Asphalt is a typical viscoelastic material with characteristics of both Hookean elasticity and Newtonian viscosity. The viscoelastic model also has elastic and viscous components, which is general modelled by combining spring and dashpot. Different models study the viscoelastic properties of asphalt materials, such as Maxwell, Kelvin, Burgers models, etc. The Maxwell model is a simple linear model that combines both Hookean springs and Newtonian dashpots in series, while the Kelvin model is a combination of Hookean springs and Newtonian dashpots in parallel. Neither the Maxwell nor the Kelvin model could fully describe the characteristics of viscoelastic materials. Thus, a Burgers model (as shown in Figure 2) is developed as a combination of Maxwell and Kelvin models in series, which is a four-element model, indicating elastic deformation, viscous flow and viscoelastic deformation [22,23].

The constitutive equation of the Burgers model is given in differential form as:

(4) $$\sigma +p_{1}\dot{\sigma }+p_{2}\ddot{\sigma }=q_{1}\dot{\varepsilon }+q_{2}\ddot{\varepsilon }$$

where *p*_1_ = (*η*_1_*E*_1_ + *η*_1_*E*_2_ + *η*_2_*E*_1_)/(*E*_1_*E*_2_), *p*_2_ = *η*_1_*η*_2_/*E*_1_*E*_2_, *q*_1_ = *η*_1_, *q*_2_ = *η*_1_*η*_2_/*E*_2_.

An applied constant stress *σ* = Δ(*t*)*σ*_0_, is introduced into Equation (4). Applying the Laplace transform can lead to Equation (5), in which *s* is Laplace operator.

(5) $$\sigma_{0}/s+p_{1}\sigma_{0}+p_{2}s\sigma_{0}=q_{1}s\bar{\varepsilon }(s)+q_{2}s^{2}\bar{\varepsilon }(s)$$

Then Equation (5) could be solved as:

(6) $$\bar{\varepsilon }(s)=\sigma_{0}\left[\frac{1}{s^{2}(q_{1}+q_{2}s)}+\frac{p_{1}}{s(q_{1}+q_{2}s)}+\frac{p_{2}}{q_{1}+q_{2}s}\right]$$

Taking the inverse Laplace transform, the creep strain versus time is given by:

(7) $$\varepsilon (t)=\sigma_{0}\left[\frac{1}{E_{1}}+\frac{t}{\eta_{1}}+\frac{1}{E_{2}}\left(1-e^{-E_{2}t/\eta_{2}}\right)\right]$$

where *E*_1_, *E*_2_, *η*_1_, *η*_2_ are viscoelastic constants, which could be determined through the fitting creep test.

For the Burgers model, *E*_1_ is the modulus of immediate elasticity Burgers model, and a higher value of *E*_1_ will lead to a larger resistance to deformation while loading, as well as a better recovery capacity after unloading. *E*_2_ is the modulus of delayed elasticity Burgers model, preventing the growing deformation of dashpot in the Kelvin model. *η*_1_ is the coefficient of viscosity Burgers model and related with permanent deformation after unloading. *η*_2_ is the coefficient of elastic delay viscosity Burgers model and corresponding viscoelastic deformation would be fully recovered with recovery time. In addition, retardation time is defined as *τ* = *η*_2_/*E*_2_, and a higher value of *τ* indicates that the asphalt material is close to viscous deformation and the recovery time of viscoelastic deformation after unloading is longer.

#### 2.3.3. Uniaxial Compression Failure and Static Creep Tests

(1) Uniaxial Compression Failure Test

Uniaxial compression failure test (Jinli testing technology Co., Ltd, Changchun, China) is adopted to study the stress-strain relationship, which could be calculated through the compression strength of specimens under a constant loading rate [24]. In this study, the uniaxial compression failure tests of asphalt mortar and mixture were conducted in accordance with previous research [25,26]. Asphalt mixes specimens were immersed in a water bath for 4 h at the test temperature. Then a loading with a constant speed of 50 mm/min was applied on specimens and a stress–strain curve was plotted following the test. The compression failure stress, failure strain and secant modulus (i.e., the ratio of failure stress to failure strain) were taken as further comparative analysis parameters [24].

(2) Uniaxial Compression Static Creep Test

The viscoelastic properties of asphalt materials can be determined through the creep test and more details can be referred to previous research [25,26]. Before uniaxial compression static creep test (Cooper Research Technology Ltd., Ripley, UK), the stress level for asphalt mortar and mixture were firstly chosen by using uniaxial compression failure test. Then a servo-pneumatic universal testing machine was employed to conduct the uniaxial compression static creep test at a fixed stress level and creep time for asphalt mortar and mixture, respectively. Due to different asphalt contents in asphalt mortar and mixture, different test temperatures were selected to reduce the error caused by temperature, in which the test temperatures were set as 30 °C for asphalt mortar and 50 °C for asphalt mixture [25,26]. Subsequently, the deformations of asphalt mortar and mixture could be measured using two LVDTs (Linear Variable Differential Transformers) and corresponding creep curves would be also obtained at different test temperatures.

At the stress level is *σ* = *σ*_0_, the creep compliance of asphalt materials is defined as follows:

*J*(*t*) = *ε*(*t*)/*σ*_0_ (8)

where *J*(*t*) is creep compliance; *ε*(*t*) is creep strain; *σ*_0_ is constant stress.

#### 2.3.4. Simplex Lattice Design (SLD)

**Figure 3:**
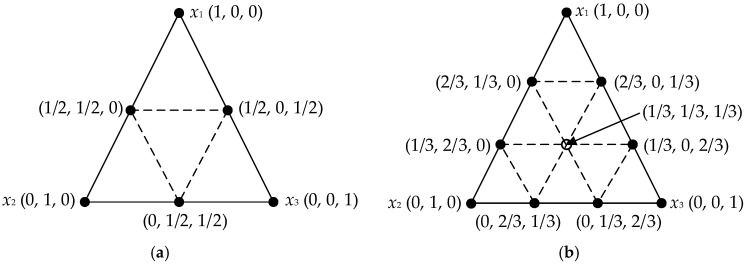
Three-ingredient simplex-lattice coordinate systems: (**a**) [3, 2] SLD; (**b**) [3, 3] SLD.

SLD is a common mixture design method for optimizing the proportions of the ingredients in the mixture by combining the mathematical theory, statistical analysis and experimental design [27]. A [*q*, *m*] simplex lattice represents *q* components consisting of points defined by the following coordinate settings in Equations (9) and (10): a “standardized” or “normalized” simplex coordinate is established and generally written as *x_i_*, the proportions assumed each component takes the (*m* + 1) equally spaced values (*x_i_*) from 0 to 1 and component proportions should be expressed as fractions with a sum (*x_i_*) of one. Figure 3 shows the three-ingredient equilateral triangular simplex-lattice coordinate systems with quadratic and cubic orders.

*x_i_* = 0, 1/*m*, 2/*m*, …, 1, (*i* = 1, 2, …, *q*) (9)

(10) $$\sum_{i=1}^{q}x_{i}=1$$

where *q* is the number of ingredients in a mixture, *m* is usually called the degree of the simplex lattice, *x_i_* is the fractional proportion of the *i*th ingredient in the [*q*, *m*] simplex lattice.

Based on the relationship between mixture components and property responses of asphalt mixes, a regression equation can be fitted to the experimental data at the points of a [*q*, *m*] simplex lattice, which is expressed in the terms of the following polynomial equation:

(11) $$y_{\left[q,m\right]}=\beta_{0}+\sum_{i=1}^{q}\beta_{i}x_{i}+\sum_{i\leq j}^{q}\beta_{ij}x_{i}x_{j}+\sum_{i\leq j\leq k}^{q}\beta_{ijk}x_{i}x_{j}x_{k}+\cdots$$

where *y*_[*q*, *m*]_ is the response, *β*_0_, *β_i_*, *β_ij_*, *β_ijk_* are the regression coefficients, *x_i_*, *x_j_*, *x_k_* are the fractional proportion of the ingredients in the mixture.

In this study, the simplex lattice of [3, 2] shown in Figure 3a was used to investigate the effects of the proportions of three mixture components on the viscoelastic properties of asphalt mixes using Design-Expert 8.0 software (Stat-Ease Inc., Minneapolis, MN, USA). The samples of asphalt mixes can be prepared through mixing the three ingredients at different proportions. The independent variable factors are the percentages of basalt (*X*_1_), andesite (*X*_2_) and pebble/river sand (*X*_3_), respectively. The high-temperature viscoelastic properties are the modulus of immediate elasticity Burgers model (*E*_1_), coefficient of viscosity Burgers model (*η*_1_) and retardation time (*τ*), as dependent variables. The designed experimental proportion need ten groups, which include three pure component treatments, three two-component mixtures and four “augment design”. Table 3 lists the proportions of three components in asphalt mixes. And the quadratic order canonical polynomial equation derived from Equation (11) can be given as:

*y* = *β*_1_*x*_1_ + *β*_2_*x*_2_ + *β*_3_*x*_3_ + *β*_12_*x*_1_*x*_2_ + *β*_23_*x*_2_*x*_3_ + *β*_13_*x*_1_*x*_3_ (12)

where *y* is the response, *β*_1_, *β*_2_, *β*_3_, *β*_12_, *β*_23_, *β*_13_ are the regression coefficients for linear and non-linear terms.

## 3. Results and Discussion

### 3.1. Results of Morphological Characteristics of Fine and Coarse Aggregates

Due to different morphological characteristics of aggregates with different particle sizes or various combinations, it is necessary to unify them in order to analyze their influence. *Composite index* for fine and coarse aggregates is adopted to account for three morphological characteristics, i.e., roundness, perimeter index and erosion-dilation area ratio, which is given in Equation (13) [14].

(13) $$Composite\;index=\sum_{i=1}^{n}[(a_{i})(index_{i})]/\sum_{i=1}^{n}\left(a_{i}\right)$$

where *Composite index* includes composite roundness, composite perimeter index, and erosion-dilation area ratio for fine and coarse aggregates abbreviated as *FR*, *CR*, *FPI*, *CPI*, *FEDR*, *CEDR*, respectively. *a_i_* is the gradation percentage of the *i*th aggregate. *index_i_* is morphological characteristics index (*R*, *PI* and *EDR*) of the *i*th aggregate. The results of *Composite index* for fine aggregate in asphalt mortar (abbreviated as F1~F10) and coarse aggregate in asphalt mixture (abbreviated as C1~C10) are listed in Table 4.

### 3.2. Analysis of Fine Aggregate Characteristics for Viscoelastic Properties of Asphalt Mortar Using SLD

#### 3.2.1. Uniaxial Compression Failure Test Results

**Figure 4:**
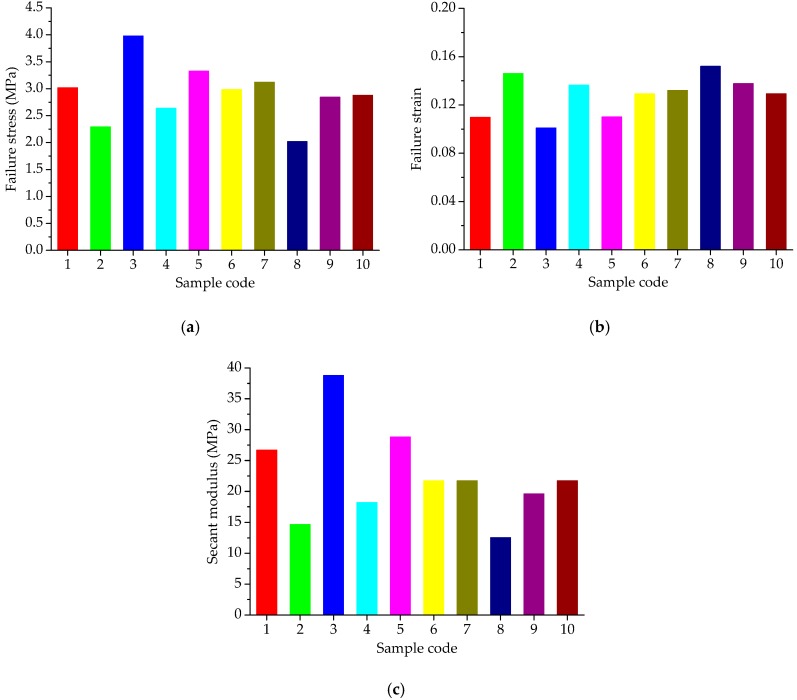
Failure results of asphalt mortars: (**a**) Failure stress; (**b**) Failure strain; (**c**) Secant modulus.

The uniaxial compression failure tests of asphalt mortar were performed at 30 °C, and the speed of applied loading was 50 mm/min. According to the recorded relation curve between force and displacement, the maximum stress, corresponding strain and the ratio of the two were obtained as the compression failure stress, failure strain and secant modulus. The uniaxial compression failure test results are plotted in Figure 4.

As shown in Figure 4a, asphalt mortars with various fine aggregate morphologies have obvious different mechanical properties when subjected to a constant strain-rate loading. Sample F3 has the highest failure stress, followed by F5; the failure stress of F8 is the lowest. In general, a higher failure stress is preferable due to better bearing capacity. Based on the stress results, it can be considered that F3 has a better bearing capacity, and followed by F5, the bearing capacity of F8 is the lowest. As for the failure strain in Figure 4b, it illustrates the deformation characteristics of asphalt mortars while loading, which has the opposite trend with the failure stress in Figure 4a. Sample F8 has the largest failure strain, whereas the failure strain of F3 is the smallest among all the asphalt mortars. The failure strain indicates the deformation before the asphalt material being broken, which could help reduce the occurrence of crack at low temperature, but would be easy to produce a greater permanent deformation at high temperatures. Secant modulus is the ratio of failure stress to failure strain, comprehensively reflecting the compatibility of deformation. Normally, a higher secant modulus stands for the better compatibility of deformation and anti-compression failure performance. Previous research has found that the shape and texture characteristics of aggregates have a strong correlation with the resistance of asphalt mixes to permanent deformation measured using different wheel tracking devices [14,28]. The failure results are consistent with the morphological characteristics results of aggregates. Thus, it could be considered that asphalt mixes with complex morphological characteristics have higher failure stress, smaller strain, and higher secant modulus. The variation trend of secant modulus results observed in Figure 4c is consistent with the trend of failure stress in Figure 4a, in which sample F3 has the highest secant modulus, followed by F5; the secant modulus of F8 is the lowest among all the asphalt mortars. As the designed experimental proportion listed in Table 3, asphalt mortar F3 was prepared by andesite manufactured sand; basalt manufactured sand was used for F5 and F8 was made by river sand. Thus, it is evident that manufactured sands can improve the anti-compression failure performance of asphalt mortar and the asphalt mortar made by natural sands has the largest failure strain.

#### 3.2.2. Uniaxial Compression Creep Test Results Based on SLD

**Figure 5:**
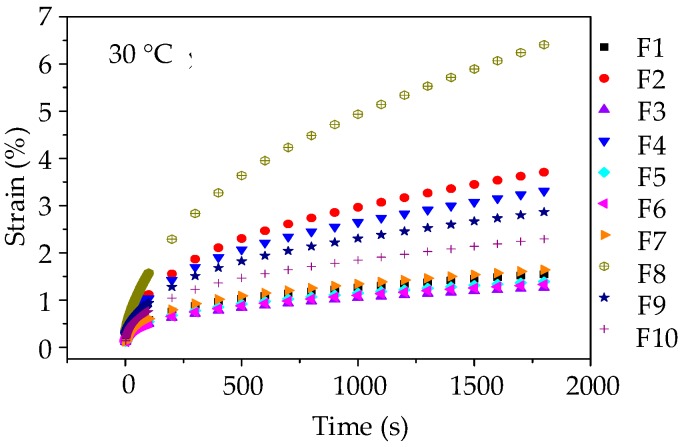
Creep strain-time curves of asphalt mortars at 30 °C.

Before the uniaxial compression static creep test, the uniaxial compression failure test was conducted to determine an appropriate stress level for asphalt mortar. Figure 4a shows the range of failure stress, i.e., 2.07 MPa~4.03 MPa for asphalt mortars at 30 °C. Thus, the applied stress level of 0.2 MPa was kept constant for the uniaxial compression creep test. Then a preconditioning stress of 5% loading was applied to asphalt mortar samples for 90 s. Subsequently, a servo-pneumatic universal testing machine was adopted to apply a stress-controlled uniaxial compressive loading for 1800 s at 30 °C. Figure 5 compares the creep strain-time curves for 10 groups of asphalt mortars at 30 °C.

As illustrated in Figure 5, the creep deformations of asphalt mortars increase gradually with the loading time increasing. Besides, it could be observed that at the same loading time, the creep strains of 10 groups of asphalt mortars are ranked as F8 > F2 > F4 > F9 > F10 > F7 > F1 > F5 > F6 > F3. A larger creep strain means a worse anti-deformation performance, that is, F3 has the best anti-deformation performance and the anti-deformation performance of F8 is the worst. Thus, andesite manufactured sand could improve the anti-deformation performance of asphalt mortar compared with river sand.

As different fine aggregates lead to different viscoelastic performances of asphalt mortars, it is necessary to quantitatively analyze the influence of morphological characteristics of fine aggregates on viscoelastic performances of asphalt mortar. The Burgers model (as shown in Figure 2) was then adopted to fit with the creep strain-time curves of asphalt mortars in order to obtain viscoelastic parameters (i.e., *E*_1_, *η*_1_ and *τ*). Table 5 details the viscoelastic responses of asphalt mortars, in which all the coefficients of determination (*R*^2^) are more than 0.98, indicating that the fitted Burgers models can well describe the creep characteristics of asphalt mortars.

#### 3.2.3. Statistical Analysis and Discussion

##### Analysis of Modulus of Immediate Elasticity Burgers Model (*E*_1_)

According to the experimental proportion design and viscoelastic responses using SLD, the analysis of variance (ANOVA) was adopted to determine the regression model and evaluate the statistical significance of independent factors, i.e., basalt (*X*_1_), andesite (*X*_2_) and pebble/river sand (*X*_3_). The statistical significance level was chosen as 0.05, that is, models and independent factors can be considered significant when the *p*-value falls below 0.05. The ANOVA results for modulus of immediate elasticity Burgers model (*E*_1_) are listed in Table 6, which shows the sum of squares, degree of freedom (DF), mean square, Fisher’s test value (*F*-value), and probability “Prob > *F*-value” (*p*-value).

Based on the ANOVA results in Table 6, the polynomial model of modulus of immediate elasticity Burgers model (*E*_1_) is demonstrated in detail. The linear terms are identified as the significant terms of *E*_1_. Thus, the quadratic order polynomial equation for *E*_1_ can be finally established as:

*Y*_1_ = 94.60*X*_1_ + 99.86*X*_2_ + 36.30*X*_3_ (14)

**Figure 6:**
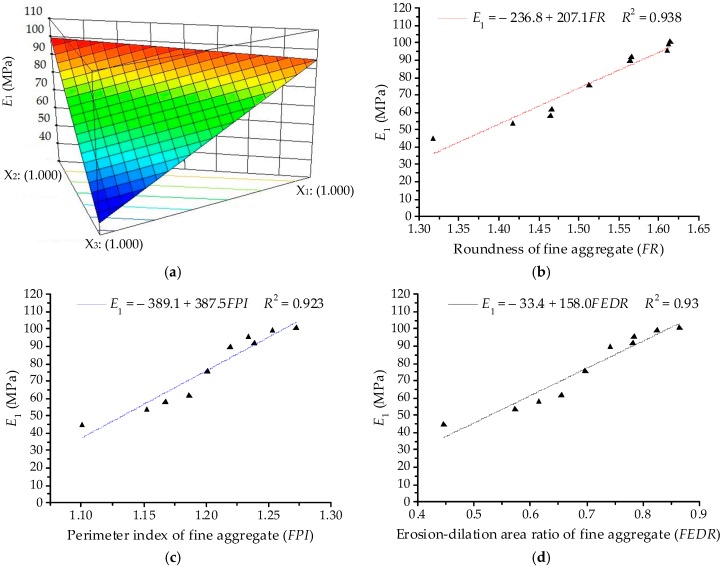
Relationships between *E*_1_ and fine aggregates: (**a**) Response surface plot for component proportion and *E*_1_; (**b**) *FR* and *E*_1_; (**c**) *FPI* and *E*_1_; (**d**) *FEDR* and *E*_1_.

Subsequently, Figure 6a illustrates the three-dimensional (3D) response surface plots for modulus of immediate elasticity Burgers model (*E*_1_), which is plotted by fitting the quadratic polynomial to reveal the effects of the component proportion on *E*_1_. It is clear from Figure 6a that *E*_1_ of asphalt mortars presents a decreasing trend when the proportion of river sand (*X*_3_) increases, whereas the proportions of basalt (*X*_1_) and andesite (*X*_2_) have opposite effects on *E*_1_. In order to quantitatively analyze the effects of fine aggregate morphologies on viscoelastic performances of asphalt mortar, morphological characteristics (i.e., *FR*, *FPI* and *FEDR*) are regarded as independent variables and the relationships of linear regression for *E*_1_ are illustrated in Figure 6b,c. The values of *E*_1_ increase with the increase of morphological characteristics of fine aggregates, for which there are positive correlations among these variables and the correlation coefficient values *R*^2^ are above 0.92. The linear regression models show a strong correlation with test results, indicating that the linear regression models are efficient in characterizing their relationship. A higher value of *E*_1_ stands for a larger resistance to deformation on loading, as well as a better recovery capacity after unloading. Thus, fine aggregates with complex morphological characteristics could improve the anti-deformation performance of asphalt mortar.

##### Analysis of Coefficient of Viscosity Burgers Model (*η*_1_)

The ANOVA results for coefficient of viscosity Burgers model (*η*_1_) are listed in Table 7 based on SLD. The linear terms are identified as the significant terms of *η*_1_. Thus, the quadratic order polynomial equation for *η*_1_ can be finally established as:

*Y*_2_ = 49.07*X*_1_ + 55.24*X*_2_ + 4.24*X*_3_ (15)

**Figure 7:**
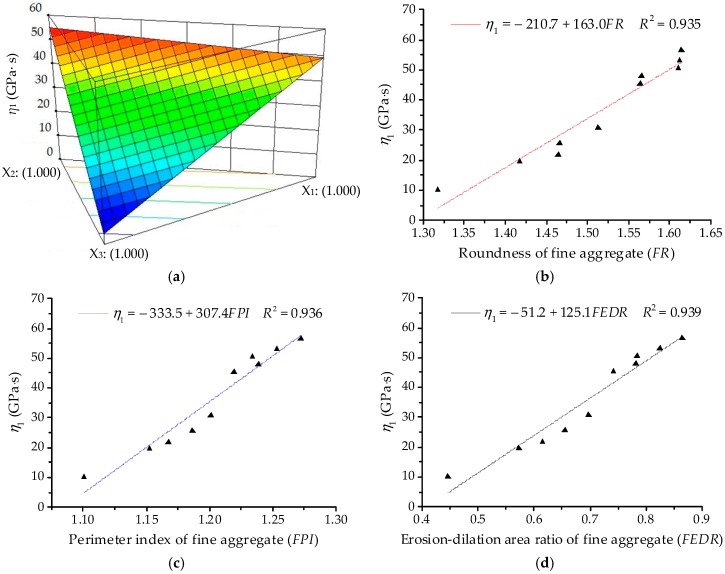
Relationships between *η*_1_ and fine aggregates: (**a**) Response surface plot for component proportion and *η*_1_; (**b**) *FR* and *η*_1_; (**c**) *FPI* and *η*_1_; (**d**) *FEDR* and *η*_1_.

As illustrated in Figure 7a, the 3D response surface plots for coefficient of viscosity Burgers model (*η*_1_) can be obtained by fitting the quadratic polynomial to reveal the effects of component proportion on *η*_1_. Figure 7b,c illustrate the positive correlation relationships of linear regression between *η*_1_ and morphological characteristics with *R*^2^ more than 0.93. Coefficient *η*_1_ shown in Figure 7 presents a similar trend with modulus of immediate elasticity Burgers model (*E*_1_) in Figure 6. *η*_1_ of asphalt mortars presents a decreasing trend when the proportion of river sand (*X*_3_) increases, whereas the proportions of basalt (*X*_1_) and andesite (*X*_2_) have similar effects on *η*_1_. The larger the viscosity coefficient *η*_1_, the smaller the permanent deformation. It also verifies that fine aggregates with complex morphologies would improve the anti-deformation performance of asphalt mortar.

##### Analysis of Retardation Time (*τ*)

The ANOVA results for retardation time (*τ*) are listed in Table 8 based on SLD. The linear terms and non-linear terms (*X*_1_*X*_3_ and *X*_1_*X*_2_*X*_3_) are identified as the significant terms of *τ*. Thus, the quadratic order polynomial equation for *η*_1_ can be finally established as:

*Y*_3_ = 105.52*X*_1_ + 106.11*X*_2_ + 210.16*X*_3_ + 50.52*X*_1_*X*_3_ − 349.97*X*_1_*X*_2_*X*_3_ (16)

**Figure 8:**
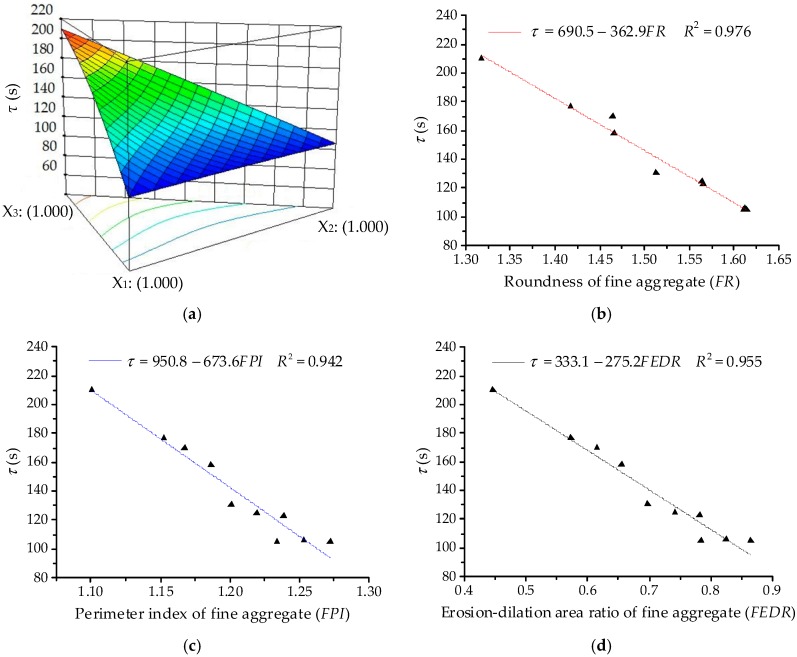
Relationships between *τ* and fine aggregates: (**a**) Response surface plot for component proportion and *τ*; (**b**) *FR* and *τ*; (**c**) *FPI* and *τ*; (**d**) *FEDR* and *τ*.

As illustrated in Figure 8a, the 3D response surface plots for retardation time (*τ*) were also obtained by fitting the quadratic polynomial to reveal the effects of component proportion on *τ*. *τ* of asphalt mortars presents a decreasing trend with increase in the proportion of basalt (*X*_1_) and andesite (*X*_2_), whereas the proportions of river sand (*X*_3_) have the opposite effect on *τ*. Figure 8b,c illustrate a negative correlation of linear regression between *τ* and morphological characteristics with *R*^2^ more than 0.94. It clearly shows that the retardation time of asphalt mortar decreases with increase in morphological characteristics of fine aggregates. As the retardation time is related to the recovery time of viscoelastic deformation, fine aggregates with complex morphological characteristics help improve the deformation recovery capacity of asphalt mortar.

### 3.3. Analysis of Coarse Aggregate Characteristics for Viscoelastic Properties of Asphalt Mixture Using SLD

#### 3.3.1. Uniaxial Compression Failure Test Results

**Figure 9:**
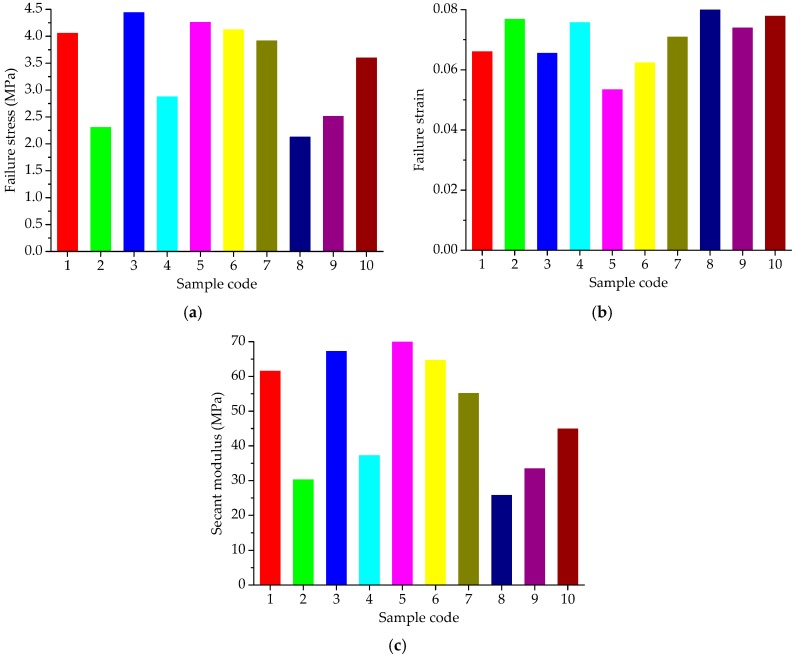
Failure results of asphalt mixtures: (**a**) failure stress; (**b**) failure strain; (**c**) secant modulus.

Figure 9 presents the uniaxial compression failure test results of asphalt mixtures at the test temperature of 50 °C. The speed of applied loading was set at 50 mm/min. As shown in Figure 9, for constant strain rate loading, different types of coarse aggregates lead to a large difference in mechanical property distribution of asphalt mixtures. Asphalt mixtures without pebbles have larger failure stress and secant modulus and those with pebbles have smaller failure strain, i.e., the failure stress and secant modulus of C3, C5 and C6 are larger than the others, while these failure strains are smaller than the other groups. The change of mechanical properties of asphalt mixtures is more related to pebble content. This is because manufactured stones (basalt stone and andesite stone) have more complex morphological characteristics than pebble and pebble is more spherical. With loading time and increasing deformation, the inlay effect of coarse aggregates becomes more and more significant. However, asphalt mixture with pebbles would cracks prematurely compared to asphalt mixture made with manufactured stones, resulting in stress reduction as well as broken specimens.

#### 3.3.2. Uniaxial Compression Creep Test Results Based on SLD

**Figure 10:**
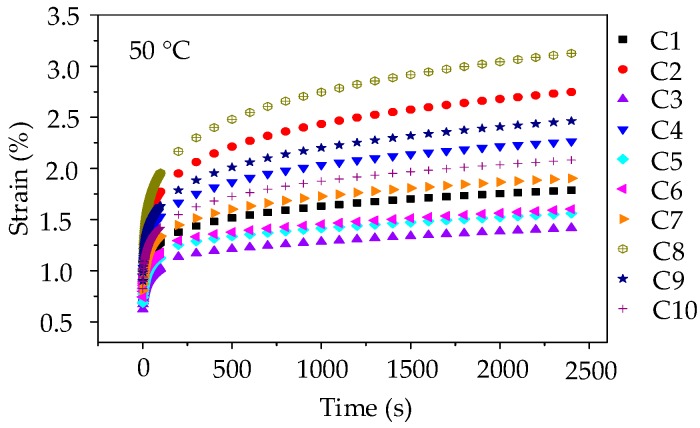
Strain-time curves of asphalt mixtures at 50 °C.

The range of failure stress for asphalt mixtures is 2.13 MPa~4.46 MPa at 50 °C. Thus, the applied stress level of 0.4 MPa was kept constant for the creep test. A preconditioning stress of 5% loading was also applied to asphalt mixture specimens for 90 s. Subsequently, a servo-pneumatic universal testing machine was adopted to apply a stress-controlled uniaxial compressive loading for 2400 s at 50 °C. Figure 10 compares the creep strain for 10 groups of asphalt mixtures.

As illustrated in Figure 10, it can be observed that at the same loading time, the anti-deformation performance of asphalt mixture prepared by manufactured stones (basalt stone and andesite stone) is better than asphalt mixture with pebble, and the creep strains of 10 groups of asphalt mixtures at any test temperature are ranked as C8 > C2 > C9 > C4 > C10 > C7 > C1 > C6 > C5 > C3. Besides, it is worth noting that when compared with the creep curves of asphalt mortars shown in Figure 5, the creep strains of asphalt mixtures are still lower under the condition that stress level is twice, test temperature is 20 °C higher, and creep time is 600 s longer. This observation fully demonstrated that the skeleton effect of coarse aggregates could effectively enhance the anti-deformation performance of asphalt mixture. Thus, coarse aggregates with complex morphological characteristics could improve the anti-deformation performance of asphalt mixture.

Similarly, the viscoelastic parameters of Burgers model were adopted to further quantitatively investigate the influence of morphological characteristics of coarse aggregates. Table 9 details the viscoelastic responses of asphalt mixtures, in which all the coefficients of determination (*R*^2^) are more than 0.97, indicating that the fitted Burgers models can well describe the creep characteristics of asphalt mixtures.

#### 3.3.3. Statistical Analysis and Discussion

##### Analysis of Modulus of Immediate Elasticity Burgers Model (*E*_1_)

The ANOVA results for modulus of immediate elasticity Burgers model (*E*_1_) are listed in Table 10. The significant terms of *E*_1_ are identified as linear terms and non-linear terms (*X*_1_*X*_2_, *X*_1_*X*_3_ and *X*_2_*X*_3_). Thus, the quadratic order polynomial equation for *η*_1_ can be finally established as:

*Y*_1_ = 48.60*X*_1_ + 53.63*X*_2_ + 37.16*X*_3_ − 20.29*X*_1_*X*_2_ − 20.84*X*_1_*X*_3_ − 37.97*X*_2_*X*_3_ (17)

**Figure 11:**
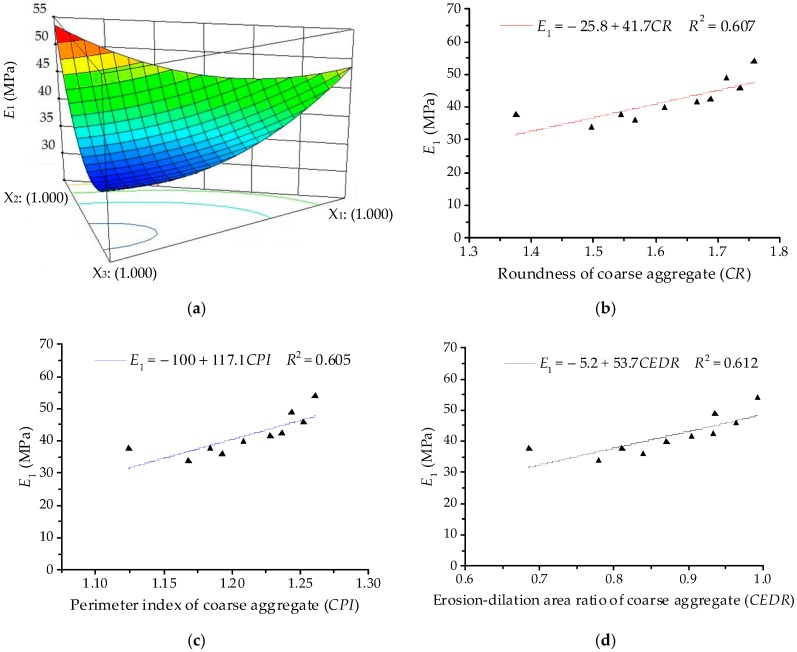
Relationships between *E*_1_ and coarse aggregates: (**a**) Response surface plot for component proportion and *E*_1_; (**b**) *CR* and *E*_1_; (**c**) *CPI* and *E*_1_; (**d**) *CEDR* and *E*_1_.

Figure 11 illustrates the relationships among modulus of immediate elasticity Burgers model (*E*_1_), component proportion, and morphological characteristics (i.e., *CR*, *CPI* and *CEDR*). Coarse aggregates of various components present a similar trend with fine aggregates, *E*_1_ of asphalt mixtures presents a decreasing trend with increase in the proportion of river sand (*X*_3_). The values of *E*_1_ increase with increase in morphological characteristics of coarse aggregates, for which there are positive correlations among these variables; the correlation coefficient values *R*^2^ are around 0.6. This means a relatively insignificant influence of coarse aggregates on *E*_1_. Morphologies of coarse aggregates are generally more related to the resistance to deformation while loading, but they present a relatively less and discretized influence on viscoelastic properties of asphalt mixture.

##### Analysis of Coefficient of Viscosity Burgers Model (*η*_1_)

The ANOVA results for coefficient of viscosity Burgers model (*η*_1_) are listed in Table 11. The significant terms of *η*_1_ are identified as linear terms and non-linear terms (*X*_1_*X*_3_ and *X*_2_*X*_3_). Thus, the quadratic order polynomial equation for *η*_1_ can be finally established as:

*Y*_2_ = 291.93*X*_1_ + 324.37*X*_2_ + 161.80*X*_3_ − 279.28*X*_1_*X*_3_ − 394.81*X*_2_*X*_3_ (18)

**Figure 12:**
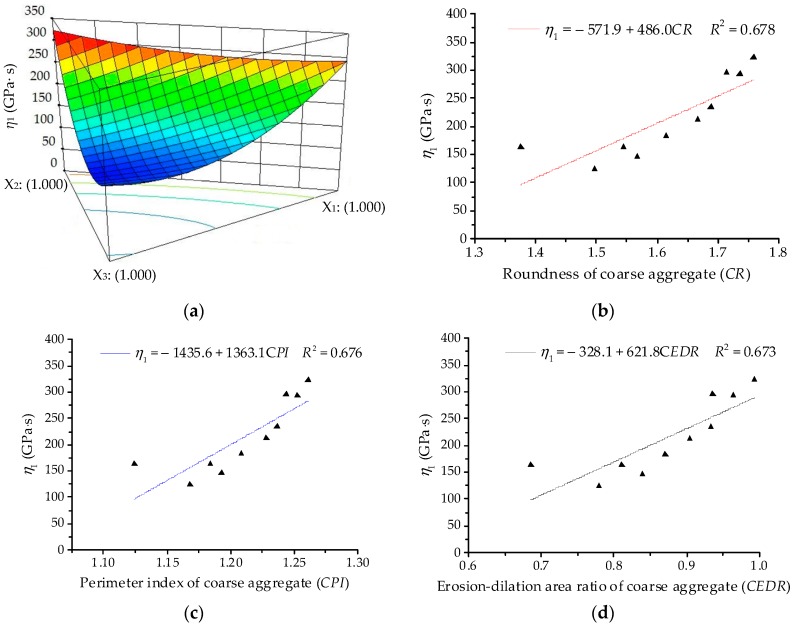
Relationships between *η*_1_ and coarse aggregates: (**a**) Response surface plot for component proportion and *η*_1_; (**b**) *CR* and *η*_1_; (**c**) *CPI* and *η*_1_; (**d**) *CEDR* and *η*_1_.

As illustrated in Figure 12, the 3D response surface and linear regression relationships are plotted for coefficient of viscosity Burgers model (*η*_1_). For coarse aggregates, asphalt mixtures with various components present a similar variation trend of *η*_1_ with asphalt mortars. However, the correlation coefficient values *R*^2^ of linear regression become relatively smaller. Morphological characteristics of coarse aggregates could improve permanent deformation to some extent. Due to the viscoelastic property of asphalt, it presents a relatively less and discretized influence on the viscoelastic properties of asphalt mixture.

##### Analysis of Retardation Time (*τ*)

The ANOVA results for retardation time (*τ*) are listed in Table 12. The linear terms are identified as the significant terms of *τ*. Thus, the quadratic order polynomial equation for *η*_1_ can be finally established as:

*Y*_3_ = 77.66*X*_1_ + 77.20*X*_2_ + 81.55*X*_3_ (19)

**Figure 13:**
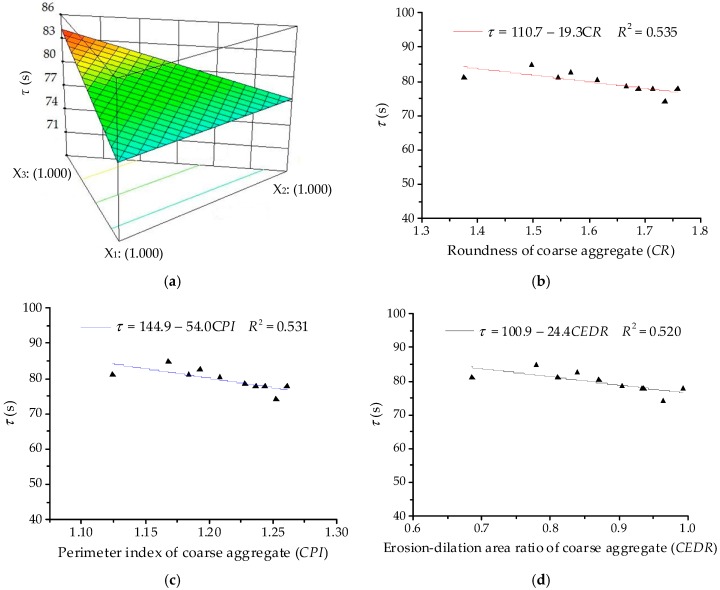
Relationships between *τ* and coarse aggregates: (**a**) Response surface plot for component proportion and *τ*; (**b**) *CR* and *τ*; (**c**) *CPI* and *τ*; (**d**) *CEDR* and *τ*.

As illustrated in Figure 13, the 3D response surface and linear regression relationships are plotted for retardation time (*τ*). It can be seen that correlation coefficient values *R*^2^ are much lower than 0.54, showing that there is no clear linear correlation between the retardation time of asphalt mixture and morphological characteristics of coarse aggregates. In fact, the retardation time of asphalt mixtures generally decreases with increase in morphological characteristics. This is expected because the viscoelastic deformation in the creep process mainly occurs in asphalt mortar, which bonds with coarse aggregates and fills internal voids. Hence, coarse aggregates are not as strongly correlated with the retardation time as fine aggregates.

## 4. Conclusions

This paper studied the influences of aggregate morphological characteristics on the high-temperature viscoelastic properties of asphalt mixes by using the SLD method. The experimental proportion scheme was designed for asphalt mixes with three different types of aggregates, i.e., basalt, andesite and pebble/river sand. Meanwhile, three morphological parameters were summarized to characterize shape, angularity and texture of fine and coarse aggregates. The uniaxial compression creep test was then conducted for asphalt mixes. The following conclusions can be drawn:

- According to correlation coefficients *R*^2^, morphological characteristics of fine aggregates were more correlated with the high-temperature viscoelastic properties of asphalt mixes compared to coarse aggregates. This may be because the viscoelastic deformation in the creep process mainly occurs in asphalt mortar;
- Aggregate morphological characteristics present a positive correlation relationships modulus of immediate elasticity Burgers model (*E*_1_) and coefficient of viscosity Burgers model (*η*_1_), but a negative correlation with retardation time (*τ*). Therefore, aggregates with more complex morphological characteristics are conducive to improving deformation recovery and anti-deformation of asphalt mixes;
- Basalt, andesite stones/manufactured sands have more complex morphological characteristics than pebble/river sand and can effectively improve the deformation recovery and anti-deformation of asphalt mixes;
- Asphalt mixes with manufactured aggregates have larger failure stress and secant modulus but lower failure strain. Meanwhile, uniaxial compression failure test results showed that coarse aggregates can effectively enhance the anti-deformation of asphalt mixture due to its skeleton effect.

## Figures and Tables

**Table 1:** Basic physical properties of asphalt AH-90.

Property	Measurement	Technical Criterion
Penetration @ 25 °C, 100 g, 5 s (0.1 mm)	90	80–100
Softening point (°C)	42.6	≥42
Ductility @ 15 °C, 5 cm/min (cm)	195.2	≥100
Density @ 15 °C (g/cm^3^)	1.014	–
*After TFOT*		
Mass loss (%)	0.37	±0.8
Penetration ratio @ 25 °C (%)	59	≥54

**Table 2:** Physical properties of aggregates and filler.

Property		Coarse and Fine Aggregates									Filler
		13.2	9.5	4.75	2.36	1.18	0.6	0.3	0.15	0.075	<0.075
Hydrophilic coefficient		−	−	−	−	−	−	−	−	−	0.80
Specific surface area (m^2^/g)		−	−	−	−	−	−	−	−	−	0.886
Apparent density (g/cm^3^)	Basalt	2.782	2.774	2.770	2.758	2.713	2.720	2.699	2.647	2.700	2.652
	Andesite	2.785	2.853	2.729	2.717	2.658	2.701	2.639	2.645	2.648	
	Pebble/River sand	2.656	2.644	2.645	2.636	2.624	2.606	2.635	2.698	2.597	

**Table 3:** Experimental proportion design for the [3, 2] simplex lattice design (SLD).

Mix Component	Sample Code									
	1	2	3	4	5	6	7	8	9	10
Basalt (*X*_1_)	0.167	0.167	0.000	0.500	1.000	0.500	0.667	0.000	0.000	0.333
Andesite (*X*_2_)	0.667	0.167	1.000	0.000	0.000	0.500	0.167	0.000	0.500	0.333
Pebble/River Sand (*X*_3_)	0.167	0.667	0.000	0.500	0.000	0.000	0.167	1.000	0.500	0.333

**Table 4:** Composite morphological index of fine and coarse aggregates.

Index	Sample Code									
	1	2	3	4	5	6	7	8	9	10
Asphalt Mortar (F1~F10)										
*FR*	1.5658	1.4175	1.6143	1.4641	1.6103	1.6123	1.5638	1.3178	1.4661	1.5127
*FPI*	1.2385	1.1526	1.2723	1.1673	1.2340	1.2532	1.2194	1.1005	1.1864	1.2011
*FEDR*	0.7821	0.5729	0.8645	0.6151	0.7842	0.8244	0.7419	0.4461	0.6553	0.6976
Asphalt Mixture (C1~C10)										
*CR*	1.6889	1.4974	1.7586	1.5447	1.7140	1.7363	1.6666	1.3755	1.5671	1.6144
*CPI*	1.2367	1.1682	1.2612	1.1840	1.2438	1.2525	1.2280	1.1242	1.1927	1.2085
*CEDR*	0.9329	0.7794	0.9928	0.8106	0.9355	0.9642	0.9043	0.6857	0.8392	0.8705

**Table 5:** Viscoelastic responses of SLD for asphalt mortars (F1~F10).

Response	Sample Code									
	1	2	3	4	5	6	7	8	9	10
*E*_1_ (MPa)	91.6	53.7	100.6	58.1	95.6	98.9	89.2	44.6	61.5	75.4
*η*_1_ (GPa·s)	48.1	19.5	56.7	21.9	50.7	53.1	45.3	10.1	25.6	30.8
*τ* (s)	122.3	176.9	105.4	170.1	105.4	105.9	124.2	209.9	157.9	130.6

**Table 6:** ANOVA results for modulus of immediate elasticity Burgers model (*E*_1_).

Source	Sum of Squares	DF	Mean Square	*F*-Value	*p*-Value	Significant
Model	3733.67	2	1866.84	64.23	<0.0001	Yes
Linear mixture	3733.67	2	1866.84	64.23	<0.0001	Yes
Residual	203.46	7	29.07			
Total	3937.14	9				

**Table 7:** ANOVA results for coefficient of viscosity Burgers model (*η*_1_).

Source	Sum of Squares	DF	Mean Square	*F*-Value	*p*-Value	Significant
Model	2324.53	2	1162.26	66.63	<0.0001	Yes
Linear mixture	2324.53	2	1162.26	66.63	<0.0001	Yes
Residual	122.11	7	17.44			
Total	2446.64	9				

**Table 8:** ANOVA results for retardation time (*τ*).

Source	Sum of Squares	DF	Mean Square	*F*-Value	*p*-Value	Significant
Model	11640.20	6	1940.03	189.13	0.0006	Yes
Linear mixture	11444.80	2	5722.40	557.86	0.0001	Yes
*X*_1_ *X*_2_	0.59	1	0.59	0.058	0.8255	No
*X*_1_ *X*_3_	107.80	1	107.80	10.51	0.0478	Yes
*X*_2_ *X*_3_	0.39	1	0.39	0.038	0.8584	No
*X*_1_ *X*_2_ *X*_3_	119.01	1	119.01	11.60	0.0423	Yes
Residual	30.77	3	10.26			
Total	11670.97	9				

**Table 9:** Viscoelastic responses of SLD for asphalt mixtures (C1~C10).

Response	Sample Code									
	1	2	3	4	5	6	7	8	9	10
*E*_1_ (MPa)	42.5	33.7	53.8	37.5	48.8	45.7	41.3	37.5	35.7	39.8
*η*_1_ (GPa·s)	235.5	124.2	321.9	163.6	294.9	292.3	212.6	163.6	145.5	183.3
*τ* (s)	77.6	84.6	77.7	81.1	77.8	74.0	78.5	81.1	82.7	80.5

**Table 10:** ANOVA results for modulus of immediate elasticity Burgers model (*E*_1_).

Source	Sum of Squares	DF	Mean Square	*F*-Value	*p*-Value	Significant
Model	345.93	5	69.19	42.38	0.0015	Yes
Linear mixture	231.36	2	115.68	70.86	0.0008	Yes
*X*_1_ *X*_2_	20.84	1	20.84	12.77	0.0233	Yes
*X*_1_ *X*_3_	21.98	1	21.98	13.46	0.0214	Yes
*X*_2_ *X*_3_	72.96	1	72.96	44.69	0.0026	Yes
Residual	6.53	4	1.63			
Total	352.46	9				

**Table 11:** ANOVA results for coefficient of viscosity Burgers model (*η*_1_).

Source	Sum of Squares	DF	Mean Square	*F*-Value	*p*-Value	Significant
Model	43176.22	5	8635.24	89.83	0.0003	Yes
Linear mixture	31170.32	2	15585.16	162.12	0.0001	Yes
*X*_1_ *X*_2_	266.41	1	266.41	2.77	0.1713	No
*X*_1_ *X*_3_	3947.65	1	3947.65	41.07	0.0030	Yes
*X*_2_ *X*_3_	7889.18	1	7889.18	82.01	0.0008	Yes
Residual	384.52	4	96.13			
Total	43560.74	9				

**Table 12:** ANOVA results for retardation time (*τ*).

Source	Sum of Squares	DF	Mean Square	*F*-Value	*p*-Value	Significant
Model	77.00	5	15.40	9.16	0.0260	Yes
Linear mixture	49.91	2	24.95	14.85	0.0141	Yes
*X*_1_ *X*_2_	9.28	1	9.28	5.52	0.0785	No
*X*_1_ *X*_3_	5.00	1	5.00	2.98	0.1596	No
*X*_2_ *X*_3_	12.69	1	12.69	7.55	0.0515	No
Residual	6.72	4	1.68			
Total	83.72	9				
